# MicroRNA-3646 Contributes to Docetaxel Resistance in Human Breast Cancer Cells by GSK-3β/β-Catenin Signaling Pathway

**DOI:** 10.1371/journal.pone.0153194

**Published:** 2016-04-05

**Authors:** Xiaohui Zhang, Shanliang Zhong, Yong Xu, Dandan Yu, Tengfei Ma, Lin Chen, Yang Zhao, Xiu Chen, Sujin Yang, Yueqin Wu, Jinhai Tang, Jianhua Zhao

**Affiliations:** 1 Center of Clinical Laboratory Science, Jiangsu Cancer Hospital Affiliated to Nanjing Medical University, Nanjing 210009, China; 2 Department of Oncology, Nanjing First Hospital, Nanjing 210006, China; 3 Department of General Surgery, Jiangsu Cancer Hospital, Nanjing 210009, China; 4 Department of Clinical Laboratory, Wuxi Second Hospital, Wuxi 214002, China; University of South Alabama, UNITED STATES

## Abstract

Acquisition of resistance to docetaxel (Doc) is one of the most important problems in treatment of breast cancer patients, but the underlying mechanisms are still not fully understood. In present study, Doc-resistant MDA-MB-231 and MCF-7 breast cancer cell lines (MDA-MB-231/Doc and MCF-7/Doc) were successfully established *in vitro* by gradually increasing Doc concentration on the basis of parental MDA-MB-231 and MCF-7 cell lines (MDA-MB-231/S and MCF-7/S). The potential miRNAs relevant to the Doc resistance were screened by miRNA microarray. We selected 5 upregulated miRNAs (has-miR-3646, has-miR-3658, has-miR-4438, has-miR-1246, and has-miR-574-3p) from the results of microarray for RT-qPCR validation. The results showed that expression level of miR-3646 in MDA-MB-231/Doc cells was significantly higher than in MDA-MB-231/S cells. Compared to MCF-7/S cells, miR-3646 expression was up-regulated in MCF-7/Doc cells. Further studies revealed that transfection of miR-3646 mimics into MDA-MB-231/S or MCF-7/S cells remarkably increased their drug resistance, in contrast, transfection of miR-3646 inhibitors into MDA-MB-231/Doc or MCF-7/Doc cells resulted in significant reduction of the drug resistance. By the pathway enrichment analyses for miR-3646, we found that GSK-3β/β-catenin signaling pathway was a significant pathway, in which GSK-3β was an essential member. RT-qPCR and Western blot results demonstrated that miR-3646 could regulate GSK-3β mRNA and protein expressions. Furthermore, a marked increase of both nuclear and cytoplasmic β-catenin expressions (with phosphorylated-β-catenin decrease) was observed in MDA-MB-231/Doc cells compared with MDA-MB-231/S cells, and their expression were positively related to miR-3646 and negatively correlated with GSK-3β. Taken together, our results suggest that miR-3646-mediated Doc resistance of breast cancer cells maybe, at least in part, through suppressing expression of GSK-3β and resultantly activating GSK-3β/β-catenin signaling pathway.

## Introduction

Breast cancer is one of the most commonly diagnosed types of malignant tumors among women of all racial and ethnic groups, and is expected to account for 29% of all new cancer cases among American women in 2015 [1]. Although the development of chemotherapeutic agents in recent years has significantly reduced mortality of breast cancer patients, subsequently resistance to these agents is still an obstacle [2]. Docetaxel (Doc) as a taxane compound by disrupting tumor cell mitosis has been routinely used solely or in combination with other anti-cancer drugs in the treatment of advanced or metastasis breast cancer. However, the resistance to Doc is often observed during treatment of breast cancer patients [3]. A number of studies have shown several mechanisms involved in acquiring drug resistance in breast cancer, including the alteration of drug transporters effluxed anticancer agents, DNA methylation, histone modification, activation of cell-survival pathway and/or inhibition of apoptotic pathway [4–6].

MicroRNAs (miRNAs) are a family of 19–25 nucleotides single-stranded non-coding RNA molecules that regulate the expression of target genes through binding to complementary sequences located in the 3’ untranslated regions (UTRs) of target messenger RNAs, leading to the translational repression and/or the sequence-specific degradation of their target mRNAs [7]. MiRNAs-mediated gene regulation is thought to play a crucial role in multiple fundamental biological processes, including embryonic development, cell proliferation, differentiation, apoptosis, and autophagy [8]. Importantly, aberrant miRNAs expression has been shown to relate to tumorigenesis and response of tumor cells to treatment. Several studies have demonstrated that deregulation of miRNAs are associated with drug-resistance of cancers including breast cancer [9].

Glycogen Synthase Kinase 3β (GSK-3β), a muti-substrate target protein kinase, is an essential component of the Wnt/β-catenin signaling pathway which is one of the most studied Wnt signaling pathway. This pathway plays important roles in the development of embryo and various malignancies, and has been recently reported to correlate significantly with chemoresistance [10]. In the absence of Wnt signaling, β-catenin is constitutively phosphorylated by GSK-3β on N-terminal residues, and then targeted by ubiquitination [11]. Reversely, the inhibition of GSK-3β activity contributes to the accumulation of β-catenin in the cytoplasm followed by its nuclear translocation [12]. Consequently, nuclear β-catenin associates with T-cell factor/lymphocyte enhancer factor (TCF/LEF) family of transcription factors and activates transcription of genes necessary for chemoresistance.

To explore the mechanism involved in Doc-resistance of breast cancer, we investigated miRNAs expression profile in parental sensitive breast cancer cell line and Doc-resistant subline. As an identical candidate, miR-3646 has been shown to be an important regulator responsible to Doc-resistant phenotype of breast cancer cells, and manipulates GSK-3β-dependent activation of β-catenin signaling pathway.

## Materials and Methods

### Cell lines

Human breast cancer cell lines MDA-MB-231 and MCF-7 were purchased from Cell Biology of Chinese Academy of Sciences (Shanghai, China) and Institute of Biochemistry (IBCB). Doc-resistant MDA-MB-231 and MCF-7 cell lines (MDA-MB-231/Doc and MCF-7/Doc) were successfully established in vitro by gradually increasing concentrations of Doc on the basis of parental MDA-MB-231 and MCF-7 cell lines (MDA-MB-231/S and MCF-7/S) in our laboratory. The IC50 (inhibitory concentration to produce 50% cell death) values of Doc in MDA-MB-231/S, MDA-MB-231/Doc, MCF-7/S and MCF-7/Doc cells were 2.75μM, 22.89μM, 2.83μM and 45.58μM, respectively.

To study the drug-mediated cell phenotypic modification, the selected drug-resistant cells were cultured in drug-free media for two weeks to remove the drug alone effect. In addition, untreated sensitive cells were used as control to normalize the drug effect. All the cells were maintained in high glucose Dulbecco’ modified Eagle’s medium (HyClone) supplemented with 10% fetal bovine serum, 100U/ml penicillin, and 100μg/ml streptomycin at 37°C in humidified atmosphere under 5%CO_2_.

### RNA extraction and real-time quantitative PCR (RT-qPCR)

Cells in logarithmic phase were collected, and then total RNA was extracted from the cells using TRIzol Reagent (Invitrogen, Carlsbad, CA, USA). RNA was quantified using UV absorbancies at 260 and 280nm (A260/280) on Nanodrop 2000 spectrophotometry (Thermo Scientific). Formaldehyde denaturing agarose gel electrophoresis was performed to confirm the quality of RNA. RNA was reversely transcribed using the BU-Script RT Kit (Biouniquer Technology Co, Ltd, Nanjing, China) according to manufacturer's recommendations. RT-qPCR was performed using SYBR Green PCR Master Mix (Biouniquer Technology) by LightCycler^®^480 (Roche, Switzerland). Primers used to amplify miR-3646, U6 small nuclear RNA (snRNA), GSK-3β and β-catenin are listed in S1 Table. U6 and β-actin were used as internal controls to normalize miRNA and mRNA, respectively. All the primers were purchased from Springen Biotechnology (China). All experiments were carried out along with a negative control (no template) and independently repeated three times. The judgment of PCR specificity was based on melting curve analyses which showed a single peak at the product melting temperature at the end of the 45 cycles, or based on inspection of the products after agarose gel electrophoresis.

### Cell transfection and survival analysis

To manipulate the cellular levels of miR-3646, 1×10^6^ cells were mixed with miR-3646 mimics at a final concentration of 10nM or antisense inhibitors at 50nM in 100ul culture medium without serum and penicillin-streptomycin. The mixtures were added into pulse cuvettes, and electroporated by Super Electroporator NEPA 21 Type II (poring pulse: pulse voltage, 125V; pulse length, 5ms; pulse interval, 50ms; NEPAGENE, Chiba, Japan). Then, the mixtures (include 5×10^5^ cells) were rapidly plated onto 6-well plate with 2ml of complete medium per well. The transfection efficiency was determined by green fluorescence.

After transfection, the cells were plated at a density of 8×10^4^ /ml into 96-well culture plate with 100μl of complete medium per well and cultured for 24h. The cells were treated with serial dilutions of Doc for 48h. For the drug cytotoxic assays, 20μl of a 5mg/ml solution of 3-[4,5-dimethylthiazol-2-yl]-2,5-diphenyltetrazolium-bromide (MTT; Sigma, Germany) was added to the cells and incubated for 4h, and then 150μl of dimethyl sulfoxide (DMSO, Amresco, America) was added, and thoroughly mixed to ensure cell lysis. CliniBio128 (ASYS-Hitech, Austria) was used to measure the absorbance at 490nm. Three independent experiments were performed, and the IC50 value of Doc was calculated using SPSS 16.0.

### Flow cytometric analysis

Flow cytometric analysis was performed to analyze the cell apoptosis using an Annexin-V-FITC apoptosis detection kit (BD Biosciences, Franklin Lakes, NJ). Briefly, the cell lines were treated with Doc for 24h. The treated cells were washed twice with ice-cold PBS, and incubated with Annexin-V-FITC and propidium iodide (PI) for 15min in the dark at room temperature. Cellular apoptosis was quantified using a FACScan flow cytometer (BD Biosciences).

### Bioinformatics softwares for target gene prediction

The target genes of miR-3646 were predicted by TargetScan and miRDB. KEGG (Kyoto Encyclopedia of Genes and Genomes) pathway enrichment analysis was performed to compare the target genes with the whole reference gene background using web-based DAVID (http://david.abcc.ncifcrf.gov/).

### Protein isolation and western blot analysis

To determine the cellular levels of GSK-3β, β-catenin and phospho-β-catenin (P-β-catenin), the cells were washed twice with phosphate-buffered saline (PBS) at room temperature and lysed with ice-cold radio immunoprecipitation assay (RIPA) buffer (Biouniquer Technology CO, LTD. Nanjing, China) on ice for 30min. The lysed cells were centrifuged at 14000×g for 15min at 4°C to remove cell pellet. The concentrations of proteins were measured using Nanodrop 2000 spectrophotometry (Thermo Scientific).

Equal amounts of proteins were subjected to electrophoresis on 10% SDS-polyacrylamide gradient gels and then transferred to polyvinylidene fluoride (PVDF) membrane (Sigma, Germany). After blocking with 5% skim milk in 0.05% Tween20/TBS (TBST), the membranes were incubated with primary antibodies overnight at 4°C and then incubated with secondary antibodies for 1h at room temperature. The primary antibodies include anti-β-actin monoclonal antibody (1:10000, Proteintech, USA), anti-GSK-3β mouse monoclonal antibody (1:100, Santa Cruz, CA), anti-β-catenin rabbit polyclonal antibody and anti-P-β-catenin rabbit polyclonal antibody (1:1000, Cell Signaling, Danvers, MA). The secondary antibodies are horse radish peroxidase labeled goat anti-rabbit (1:2000, Cell Signaling, Danvers, MA) and goat anti-mouse (1:5000, Kangwei Ltd. Beijing, China). The membranes were developed with an ECL Substrate (Biouniquer Technology, CO, LTD).

### Immunofluorescence analysis

Cells were grown to approximately ~70% confluence on sterile coverslips, fixed with 4% paraformaldehyde for 30 min at room temperature, and then permeabilized with 0.5% Triton X-100 in PBS for 20 min on ice. After blocking with 5% BSA for 1h, cells were incubated with the primary antibody (rabbit polyclonal antibody against β-catenin, 1:200 dilution) for overnight at 4°C. Cells were then incubated with Alexa Fluor^®^594 conjugated goat anti-rabbit secondary antibody (1:250 dilution, Jackson Immuno Research) for 1h at room temperature in the dark. To ensure specificity of the results, negative controls without primary antibody or secondary antibody were included. Finally, cells were counterstained with Hoechst 33342 (1:1000 dilution), mounted with mounting medium (1:1000 dilution, Thermo Fisher Scientific) and visualized using Zeiss Axio Vert. A1 inverted fluorescent microscope (Carl Zeiss Microscopy GmbH, Germany). Images were acquired at 200× total magnification using Zeiss Zen 2012 software.

### Statistical Analysis

All the experiments were performed three independent times. Statistical analysis was performed using SPSS 16.0 statistical package. Data were presented as mean±SD (standard deviation). Student’s *t* test was used for the assessment of statistical significance of difference between two independent groups. A p value <0.05 was considered statistically significant.

## Results

### Identification of miR-3646 in breast cancer cells responsible to doc treatment

**Fig 1:**
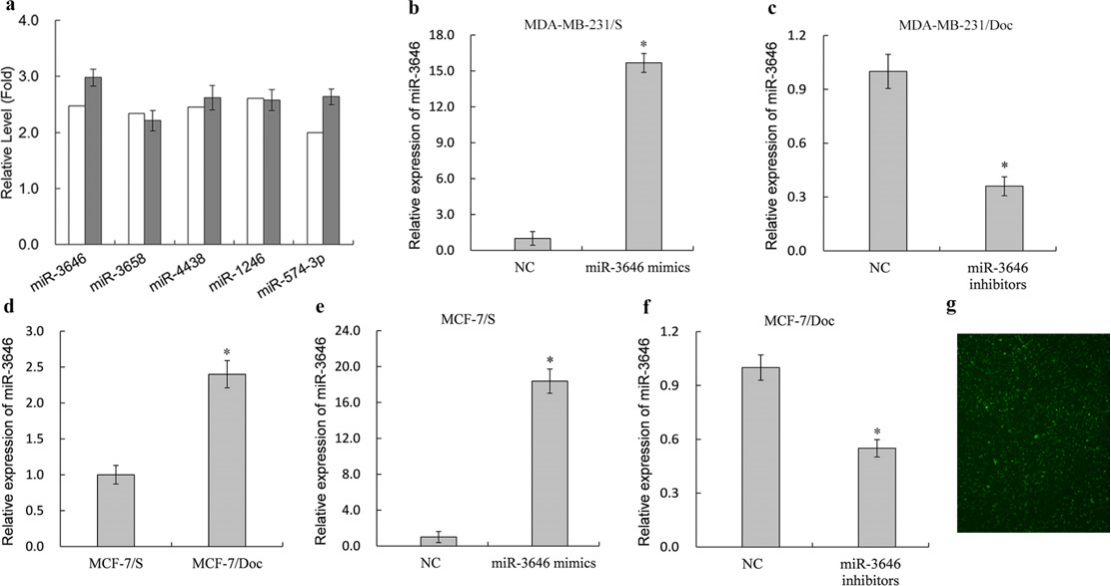
Relative expression level of miRNAs in the breast cancer cells. **a** Relative expression of 5 miRNAs in MDA-MB-231/S and MDA-MB-231/Doc cells. **b,e** Relative expression of miR-3646 in MDA-MB-231/S or MCF-7/S cells transfected with miRNA-3646 mimics and negative control of mimics (NC) (*P<0.05). **c,f** Relative expression of miR-3646 in MDA-MB-231/Doc or MCF-7/Doc cells transfected with miR-3646 inhibitors and negative control of inhibitors (NC) (*P<0.05). **d** Relative expression miR-3646 of in MCF-7/S and MCF-7/Doc cells. **g** The transfection efficiency of electroporation was determined by green fluorescence.

The miRNA expression profiles of MDA-MB-231/S and MDA-MB-231/Doc were evaluated using Affymetrix GeneChip miRNA 4.0 Array. From several differentially expressed miRNAs obtained from the microarray, we selected 5 upregulated miRNAs (has-miR-3646, has-miR-3658, has-miR-4438, miR-1246, and has-miR-574-3p) for RT-qPCR validation. The RT-qPCR results of the 5 miRNAs showed good consistency with microarray results (Fig 1A). In particular, transcript of miR-3646 in MDA-MB-231/Doc cells was up-regulated by 2.98-fold compared to MDA-MB-231/S cells. Furthermore, expression level of miR-3646 in MCF-7/Doc cells was higher than in MCF-7/S cells (Fig 1D). Hence, we selected miR-3646 to investigate its association with the sensitivity of breast cancer cells to Doc and potential mechanism. To manipulate the cellular levels of miR-3646, its mimics were transfected into MDA-MB-231/S or MCF-7/S cells. RT-qPCR analysis indicated that miR-3646 was up-regulated by 15.67-fold compared to negative control of mimics (NC) in MDA-MB-231/S cells, and increased by 18.38-fold compared to NC in MCF-7/S cells (Fig 1B and 1E). In addition, miR-3646 inhibitors were transfected into MDA-MB-231/Doc or MCF-7/Doc cells. The results showed that miR-3646 was down-regulated by 0.36-fold compared to negative control of inhibitors (NC) in MDA-MB-231/Doc cells, and decreased by 0.55-fold compared to NC in MCF-7/Doc cells (Fig 1C and 1F). As shown in Fig 1G, the transfection efficiency by the method of electroporation was greater than 90%.

**Fig 2:**
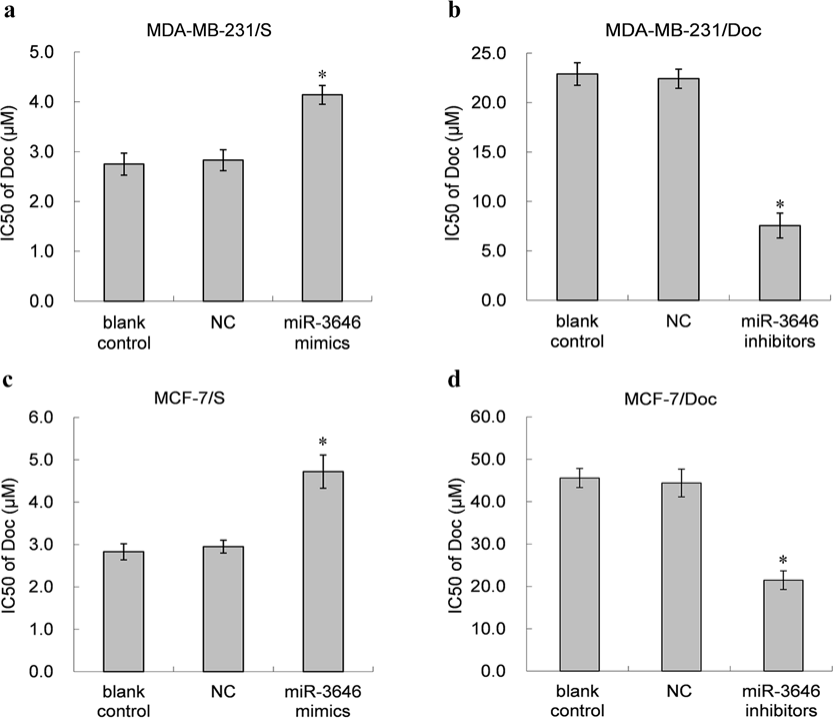
Effect of miR-3646 mimics and miR-3646 inhibitors on the sensitivity of breast cancer cell lines to Doc. **a,c** The IC50 value of Doc was determined after MDA-MB-231/S or MCF-7/S cells were transfected with miR-3646 mimics, NC or blank control for 48h using MTT assay (*P<0.05). **b,d** After MDA-MB-231/Doc or MCF-7/S cells were transfected with miR-3646 inhibitors, NC or blank control for 48h, theIC50 value of Doc was determined by MTT assay (*P<0.05).

To testify whether the levels of miR-3646 are related with drug resistance of breast cancer cells, MTT assay was performed to measure cytotoxicity of Doc after transfection of miR-3646 mimics or inhibitors. The results showed that up-regulation of miR-3646 significantly increased Doc resistance of MDA-MB-231/S or MCF-7/S cells (Fig 2A and 2C), whereas, down-regulation of miR-3646 decreased Doc resistance of MDA-MB-231/Doc or MCF-7/Doc cells (Fig 2B and 2D). The results suggest that inhibiting the expression of miR-3646 could reverse the drug resistance of breast cancer cells to Doc.

### The effect of miR-3646 on Doc-induced apoptosis

**Fig 3:**
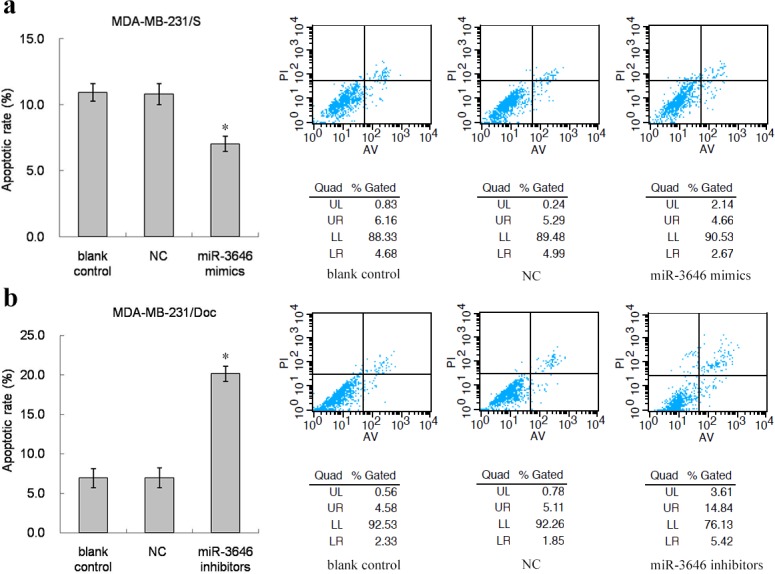
Flow cytometric analysis of apoptotic MDA-MB-231/S and MDA-MB-231/Doc cells induced by Doc. **a** The apoptotic rate and representative FACS figures of MDA-MB-231/S cells transfected with miR-3646 mimics were significantly decreased compared with NC and blank control (*P<0.05). **b** The apoptotic rate and representative FACS figures of MDA-MB-231/Doc cells transfected with miR-3646 inhibitors were significantly augmented compared with NC and blank control (*P<0.05).

**Fig 4:**
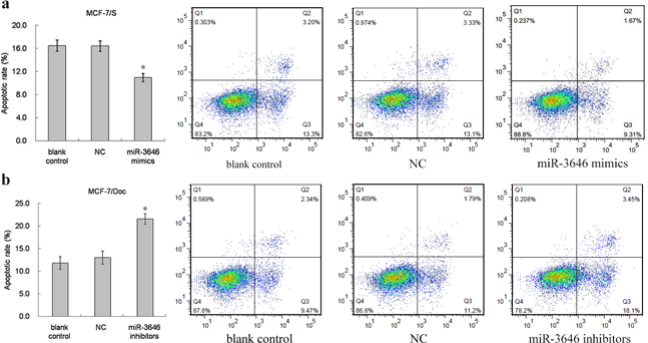
Flow cytometry assessment of apoptotic MCF-7 cells induced by Doc was determined using APC Annexin V. **a** The apoptotic rate and representative FACS figures in MCF-7/S cells transfected with miR-3646 mimics, NC and blank control (*P<0.05). **b** The apoptotic rate and representative FACS figures in MCF-7/Doc cells transfected with miR-3646 inhibitors, NC and blank control (*P<0.05).

Flow cytometric analysis was used to quantify cell apoptotic rate of breast cancer cell lines. As shown in Figs 3A and 4A, transfection with miR-3646 mimics significantly reduced apoptotic cell numbers compared to negative and blank controls. In contrast, transfection with miR-3646 inhibitors increased apoptosis compared to negative and blank controls (Figs 3B and 4B). The results indicate that the expression of miR-3646 is associated with cell apoptotic progression.

### Prediction of miR-3646 target genes

**Fig 5:**
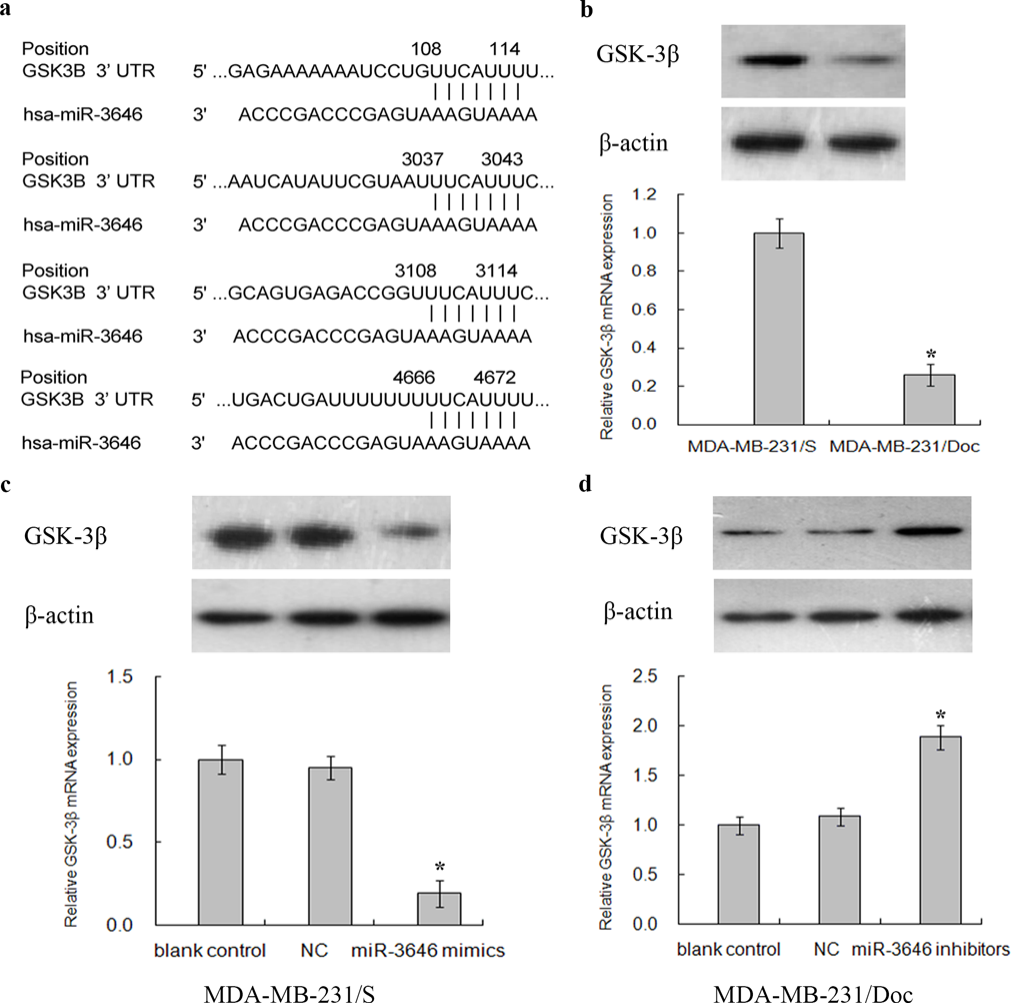
GSK-3β was a candidate target of miRNA-3646. **a** Four complementary sites of miR-3646 in GSK-3β 3’UTR were predicted by bioinformatics analysis. **b** Relative mRNA and protein expression of GSK-3β in MDA-MB-231/S and MDA-MB-231/Doc cells. **c** Relative mRNA and protein expression of GSK-3β in MDA-MB-231/S cells transfected with miR-3646 mimics, NC and blank control. **d** Relative mRNA and protein expression of GSK-3β in MDA-MB-231/Doc cells transfected with miR-3646 inhibitors, NC and blank control.

More than one thousand potential target genes of miR-3646 were predicted by TargetScan and 1602 by miRDB. KEGG pathway enrichment analysis further narrowed the predicted target genes to 582 based on a cut-off criterion that the count number larger than 2 and Bonferroni *P*-value of less than 0.05. The potential target genes are involved in 20 pathways (S2 Table). In particular, GSK-3β is a key member in the GSK-3β/β-catenin pathway, which is suspected as a major sub-pathway of "pathways in cancer"(hsa05200). Thus, we finally selected GSK-3β for further functional studies to assess the effect of miR-3646 on Doc resistance of breast cancer cells. Subsequently, 4 complementary binding sites of miR-3646 in GSK-3β 3’ UTR were identified, including positions of 108–114, 3037–3043, 3108–3114, and 4666–4672 (Fig 5A).

### Correlation of GSK-3β and miR-3646 in responsible to Doc resistance of breast cancer cells

The levels of mRNA and proteins were quantified by RT-qPCR and Western blot. GSK-3β mRNA and protein in MDA-MB-231/S cells were higher than those in MDA-MB-231/Doc cells (Fig 5B). After transfection with miR-3646 mimics, the expression levels of the GSK-3β gene in MDA-MB-231/S cells was reduced (Fig 5C). In contrast, transfection of miR-3646 inhibitors increased the levels of GSK-3β mRNA and protein in MDA-MB-231/Doc cells (Fig 5D). The correlation between miR-3646 and GSK-3β suggests that GSK-3β is a potential target of miR-3646, which negatively regulates Doc resistance of breast cancer cells.

### The subcellular localization and relative level of β-catenin in responsible to doc resistance of breast cancer cells

**Fig 6:**
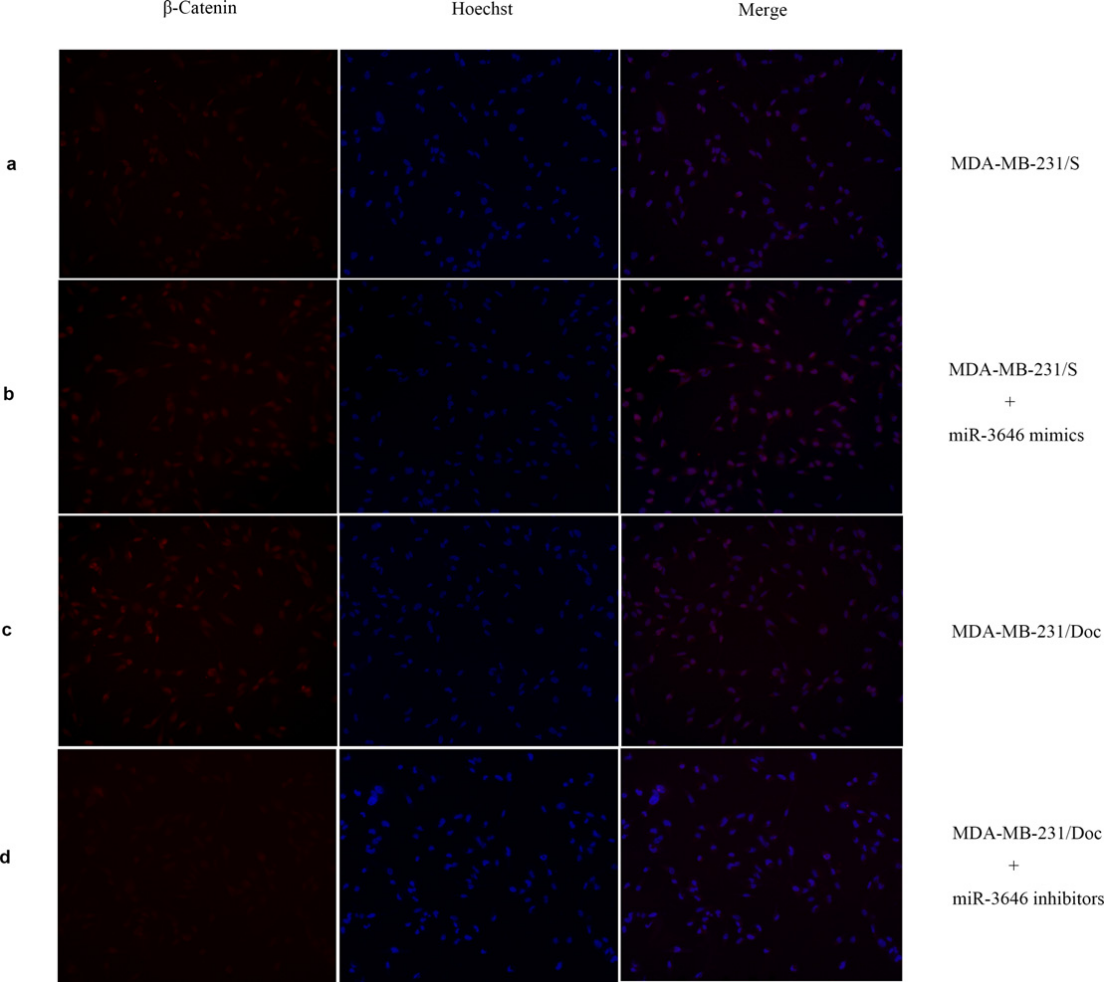
β-catenin immunofluorescence in breast cancer cells. **a,c** MDA-MB-231 cells displayed both nuclear and cytoplasmic β-catenin expression. The expression of β-catenin in MDA-MB-231/Doc cells was highly regulated as compared to the expression levels in MDA-MB-231/S cells. **b** The sensitive cells displayed increased nuclear and cytoplasmic β-catenin expression after transfection with miR-3646 mimics. **d** The resistant cells showed decreased nuclear and cytoplasmic β-catenin expression after transfection with miR-3646 inhibitors. Blue = nuclear staining; Red = β-catenin; Pink = merge.

β-catenin is a subunit of the cadherin family and acts as an intracellular signal transducer in the GSK-3β/β-catenin pathway for regulating the coordination of cell–cell adhesion. The inhibition of GSK-3β activity contributes to the accumulation of β-catenin in the cytoplasm followed by its nuclear translocation. This prompted us to assess whether miR-3646 could regulate the subcellular localization of β-catenin through targeting GSK-3β. Immunofluorescence showed that β-catenin was localized in the cytoplasm and the nuclei of MDA-MB-231 cells (Fig 6). However, a markly increased expression of β-catenin in nuclei and cytoplasm was observed in MDA-MB-231/Doc cells compared with MDA-MB-231/S cells (Fig 6A and 6C). Furthermore, transfection of miR-3646 mimics increased nuclear and cytoplasmic β-catenin expression in MDA-MB-231/S cells (Fig 6B). In contrast, transfection of miR-3646 inhibitors in MDA-MB-231/Doc cells was able to reduce nuclear and cytoplasmic β-catenin expression (Fig 6D).

**Fig 7:**
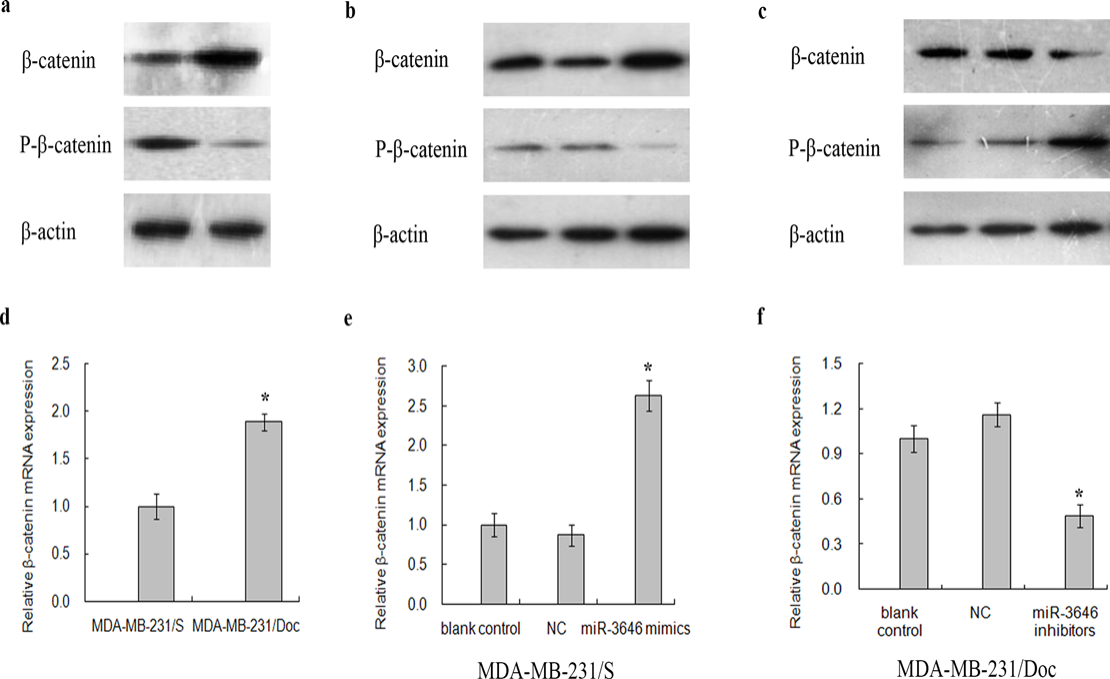
RT-qPCR and Western blot analysis of β-catenin and P-β-catenin expression in MDA-MB-231/S and MDA-MB-231/Doc cells. **a** Protein expression of β-catenin and P-β-catenin in MDA-MB-231/S and MDA-MB-231/Doc cells. **b** Protein expression of β-catenin and P-β-catenin in MDA-MB-231/S cells transfected with miR-3646 mimics, NC and blank control. **c** Protein expression of β-catenin and P-β-catenin protein in MDA-MB-231/Doc cells transfected with miR-3646 inhibitors, NC and blank control. **d** Relative mRNA expression of β-catenin in MDA-MB-231/S and MDA-MB-231/Doc cells (*P<0.05). **e** Relative mRNA expression of β-catenin in MDA-MB-231/S cells transfected with miR-3646 mimics, NC and blank control (*P<0.05). **f** Relative mRNA expression of β-catenin in MDA-MB-231/Doc cells transfected with miR-3646 inhibitors, NC and blank control (*P<0.05).

Phosphorylation of β-catenin by GSK-3β could regulate the stabilization of β-catenin. We further analyzed the expression level of β-catenin and P-β-catenin in these cells to explore whether miR-3646 could regulate them. The results showed that the expression of β-catenin in MDA-MB-231/Doc cells was up-regulated compared to MDA-MB-231/S cells (Fig 7A and 7D). Consistently, the relative expression levels of P-β-catenin protein were reduced in MDA-MB-231/Doc cells (Fig 7A). Furthermore, transfection of miR-3646 mimics increased β-catenin but decreased its phosphorylated form in MDA-MB-231/S cells, and additionally the relative expression levels of GSK-3β were consistent with the results (Fig 7B and 7E). As expected, transfection of miR-3646 inhibitors in MDA-MB-231/Doc cells reduced β-catenin but increased its phosphorylated form, which is consistent with the high levels of GSK-3β (Fig 7C and 7F). Our results suggest that miR-3646 may modulate the GSK-3β/β-catenin signaling pathway through targeting GSK-3β.

## Discussion

Breast cancer is the most frequently diagnosed cancer and the leading cause of cancer-related death among women worldwide [13]. Triple-negative breast cancer (TNBC) is a special subtype of breast cancer that is characterized by the absence of estrogen receptors (ER) and progesterone receptors (PR) and does not show over-expression of Her-2/neu (HER2) [14]. Consequently, TNBC is insensitive to clinical treatments that are currently used for effectively treating other types of breast cancer, including endocrine therapies and HER2-directed therapies [15]. Since no efficiency of regular chemotherapies as well as poor prognosis due to metastasis and relapse, TNBC has attracted a lot of scientific interests in investigation of mechanisms underlying its aggressive phenotype. Recently discovered microtubule-poisoning drug has been shown to efficiently treat TNBC, such as docetaxel and paclitaxel [16]. Although more than half of the patients treated with docetaxel achieve a good response, development of acquired resistance to docetaxel often occurs and is a notable clinical problem [7]. Thus, the present study investigated the role of miRNA-mediated gene regulation involved in Doc resistance using MDA-MB-231, a type of TNBC cell line.

It has been frequently reported that the functions of miRNAs are related to tumorigenesis and response of tumor cells to treatment [17]. Increasing evidence demonstrated that the dysregulated expressions of miRNAs were associated with chemoresistance of multiple cancers, particularly breast cancer [8]. Subsequently, miRNAs-mediated drug resistance has arisen much attention in the development of new approaches for reversible chemoresistance of cancer cells using a novel strategy of miRNA targets [18]. Previous studies demonstrated that circulating miR-3646 was highly expressed in patients with ischemic hepatitis, bladder cancer, and breast cancer [19–21]. In this study, the results showed that the increase of miR-3646 expression was associated with drug resistance of MDA-MB-231 cells to Doc, but inhibiting its expression could reverse the drug resistance of cells to Doc. Furthermore, we found the consistent results in MCF-7 breast cancer cell line.

Wnt/β-catenin signaling pathway plays important roles in cell proliferation, differentiation, tumorigenesis and chemoresistance of cancer [22]. The central member of the Wnt/β-catenin pathway is β-catenin. Aberrant β-catenin expression as determined by assessment of its subcellular location constitutes a surrogate marker of this pathway activation and has been reported in several human cancers, including colon carcinomas, melanomas, pilomatricomas, and hepatocellular carcinoma [23, 24]. Furthermore, Sokolosky *et al* have showed that inhibition of GSK-3β activity could result in drug resistance and alter sensitivity to targeted therapy in breast cancer cells [25]. The present identify miR-3646, is obviously up-regulated and consequently leads to suppression of GSK-3β in breast cancer cells, suggesting that miR-3646 may modulate the drug resistance by targeting GSK-3β. It has been reported that GSK-3β destabilizes β-catenin via phosphorylating it at Ser-33, Ser-37 and Thr-41 residues [26]. We showed that miR-3646 suppressed GSK-3β and further determined its effect on increasing β-catenin stability by reducing its phosphorylated unstable form. Consistently, up-regulation of GSK-3β resulted in the increase of phosphorylated β-catenin protein leading to β-catenin degradation. Our results suggest a mechanism by which miR-3646 contributes Doc resistance of MDA-MB-231 cells through activating GSK-3β/β-catenin pathway.

In addition, GSK-3β is also an intermediator for PI3K/AKT/mTOR signaling pathway by enhancing phosphorylation of AKT, which inactivates GSK-3β and accumulates β-catenin [27]. Tzeng *et al* recently demonstrated that the activation of Wnt/β-catenin pathway interacted with PI3K/AKT/mTOR pathway in MDA-MB-231 cells and that resistance of the cells to PI3K inhibitors was mediated by the crosstalk between Wnt/β-catenin and PI3K/AKT/mTOR pathways [15].The present study reveals that miR-3646 directly targets GSK-3β and contributes to doc resistance of the cells. Overexpression of miR-3646 may also be involved in inhibition of PI3K/AKT/mTOR pathway, which is currently under investigation respectively.

## Conclusions

In summary, we firstly demonstrates that miR-3646 contributes to drug resistance of breast cancer cells to Doc at least in part through activation of GSK-3β/β-catenin pathway by suppressing expression of GSK-3β. Our results give us an insight into mechanisms of drug resistance of breast cancer cells and provide a promising approach to enhance efficiency of chemotherapy by inhibiting miR-3646.

## Supporting Information

S1 Table Primers used for RT-qPCR. Primers of U6, miR-3646, β-actin, GSK-3β and β-catenin.
(DOC)

S2 Table KEGG pathway enrichment analysis with DAVID tool. The potential target genes are involved in 20 pathways (*P*<0.05).
(DOCX)
