# Supplementary material for: MicroRNA-3646 Contributes to Docetaxel Resistance in Human Breast Cancer Cells by GSK-3β/β-Catenin Signaling Pathway
Source: PLoS One. 2016 Apr 5;11(4):e0153194. doi: 10.1371/journal.pone.0153194 (PMC4821636; doi:10.1371/journal.pone.0153194)
Supplement: S1 Table — Primers of U6, miR-3646, β-actin, GSK-3β and β-catenin. (DOC) [file pone.0153194.s001.doc]

**Table S1 Sequences of RT primers, forward primers and reverse primers using for RT-qPCR**

| **miRNA/mRNA ID** | **RT primer(5′-3′)** | **Forward Primer (5′-3′)** | **Reverse Primer (5′-3′)** |
| --- | --- | --- | --- |
| U6 | GCGCGTGAGCAGGCTGGAGAAATTAACCACGCGCGGAACG | CGCAAGGATGACACG | GAGCAGGCTGGAGAA |
| miR-3646 | GTCGTATCCAGTGCGTGTCGTGGAGTCGGCAATTGCACTGGATACGACTGGGCTG | CCCCAAAATGAAATGAGCC | CAGTGCGTGTCGTGGAGT |
| β-actin | random primers | CACCTTCTACAATGAGCTGCGTGTG | ATAGCACAGCCTGGATAGCAACGTAC |
| GSK-3β | random primers | CGAGACACACCTGCACTCTT | TTAGCATCTGACGCTGCTGT |
| β-catenin | random primers | GCGCCATTTTAAGCCTCTCG | CCTCAGACCTTCCTCCGTC |
