# Supplementary material for: MicroRNA-3646 Contributes to Docetaxel Resistance in Human Breast Cancer Cells by GSK-3β/β-Catenin Signaling Pathway
Source: PLoS One. 2016 Apr 5;11(4):e0153194. doi: 10.1371/journal.pone.0153194 (PMC4821636; doi:10.1371/journal.pone.0153194)
Supplement: S2 Table — The potential target genes are involved in 20 pathways (P<0.05). (DOCX) [file pone.0153194.s002.docx]

**Table S2. KEGG pathway enrichment analysis with DAVID tool**

| \| Term \| Gene Count \| % \| P-Value \| Genes \| \| --- \| --- \| --- \| --- \| --- \| \| hsa04120:Ubiquitin mediated proteolysis \| 13 \| 2.43 \| 0.0004 \| VHL,UBE2D3,XIAP,RCHY1,UBE2W,UBA6,UBE2J2,CUL1,CBL,CUL3,MID1,MAP3K1,UBE2QL1 \| \| hsa04720:Long-term potentiation \| 9 \| 1.69 \| 0.0005 \| GRIN2A,PRKCA,PPP1CB,PPP1R12A,GRIA2,RAP1A,MAPK1,RPS6KA6,CACNA1C \| \| hsa05200:Pathways in cancer \| 21 \| 3.93 \| 0.0006 \| FOXO1,CHUK,VHL,GLI3,FGF2,CDK6,XIAP,MAPK1,TCF7L2,CBL,PRKCA,HDAC2,HDAC1,AXIN2,RHOA,AKT3,FGF12,GSK3B,APPL1,RARA,CCDC6 \| \| hsa04360:Axon guidance \| 12 \| 2.25 \| 0.0008 \| NFAT5，GNAI3，ROBO2，UNC5D，EPHA5，RHOA，SRGAP3，GSK3B，MAPK1，ROBO1，CXCL12，NRP1 \| \| hsa04010:MAPK signaling pathway \| 17 \| 3.18 \| 0.0027 \| CHUK，FGF2，RASGRP3，MAPK1，ZAK，CACNA1C，PPM1B，PRKCA，TAOK1，RAP1A，AKT3，MAP4K3，FGF12，MAP3K1，MAP3K2，ELK4，RPS6KA6 \| \| hsa04722:Neurotrophin signaling pathway \| 10 \| 1.87 \| 0.0071 \| RHOA，AP1A，YWHAQ，AKT3，MAP3K1，GSK3B，MAPK1，FRS2，FOXO3，RPS6KA6 \| \| hsa05215:Prostate cancer \| 8 \| 1.50 \| 0.0113 \| CREB5，FOXO1，CHUK，AKT3，GSK3B，MAPK1，TCF7L2，CREB1 \| \| hsa04530:Tight junction \| 10 \| 1.87 \| 0.0116 \| GNAI3，CASK，RAB3B，PRKCA，RHOA，AKT3，PPP2CB，PRKCE，ZAK，CLDN16 \| \| hsa04270:Vascular smooth muscle contraction \| 9 \| 1.69 \| 0.0120 \| ADCY7，PRKCA，PPP1CB，RHOA，PPP1R12A，MAPK1，PRKCE，KCNMA1，CACNA1C \| \| hsa05213:Endometrial cancer \| 6 \| 1.12 \| 0.0139 \| AXIN2，AKT3，GSK3B，MAPK1，FOXO3，TCF7L2 \| \| hsa04150:mTOR signaling pathway \| 6 \| 1.12 \| 0.0139 \| EIF4E，PRKAA2，AKT3，MAPK1，CAB39，RPS6KA6 \| \| hsa04666:Fc gamma R-mediated phagocytosis \| 8 \| 1.50 \| 0.0158 \| PRKCA，WASF1，AKT3，MAPK1，PRKCE，WASL，MARCKS，WASF3 \| \| hsa05220:Chronic myeloid leukemia \| 7 \| 1.31 \| 0.0172 \| CBL，CHUK，HDAC1，HDAC2，AKT3，CDK6，MAPK1 \| \| hsa04520:Adherens junction \| 7 \| 1.31 \| 0.0193 \| RHOA，WASF1，MAPK1，SORBS1，WASL，WASF3，TCF7L2 \| \| hsa04310:Wnt signaling pathway \| 10 \| 1.87 \| 0.0237 \| NFAT5，CXXC4，CUL1，PRKCA，AXIN2，RHOA，SFRP1，GSK3B，PPP2CB，TCF7L2 \| \| hsa04914:Progesterone-mediated oocyte maturation \| 7 \| 1.31 \| 0.0313 \| ADCY7，GNAI3，CPEB1，PPP1CB，AKT3，MAPK1，RPS6KA6 \| \| hsa04114:Oocyte meiosis \| 8 \| 1.50 \| 0.0323 \| CUL1，ADCY7，CPEB1，PPP1CB，YWHAQ，MAPK1，PPP2CB，RPS6KA6 \| \| hsa04350:TGF-beta signaling pathway \| 7 \| 1.31 \| 0.0329 \| CUL1，BMPR2，E2F4，RHOA，ACVR2A，MAPK1，PPP2CB \| \| hsa04910:Insulin signaling pathway \| 9 \| 1.69 \| 0.0331 \| FOXO1，EIF4E，CBL，PRKAA2，PPP1CB，AKT3，GSK3B，MAPK1，SORBS1 \| \| hsa04062:Chemokine signaling pathway \| 11 \| 2.06 \| 0.0340 \| ADCY7，GNAI3，CHUK，RHOA，RAP1A，AKT3，GSK3B，MAPK1，CXCL12，WASL，FOXO3 \| |
| --- | --- | --- | --- | --- | --- | --- | --- | --- | --- | --- | --- | --- | --- | --- | --- | --- | --- | --- | --- | --- | --- | --- | --- | --- | --- | --- | --- | --- | --- | --- | --- | --- | --- | --- | --- | --- | --- | --- | --- | --- | --- | --- | --- | --- | --- | --- | --- | --- | --- | --- | --- | --- | --- | --- | --- | --- | --- | --- | --- | --- | --- | --- | --- | --- | --- | --- | --- | --- | --- | --- | --- | --- | --- | --- | --- | --- | --- | --- | --- | --- | --- | --- | --- | --- | --- | --- | --- | --- | --- | --- | --- | --- | --- | --- | --- | --- | --- | --- | --- | --- | --- | --- | --- | --- | --- |
